# Predicting microbiologically defined infection in febrile neutropenic episodes in children: global individual participant data multivariable meta-analysis

**DOI:** 10.1038/bjc.2016.137

**Published:** 2016-05-26

**Authors:** Robert S Phillips, Lillian Sung, Roland A Ammann, Richard D Riley, Elio Castagnola, Gabrielle M Haeusler, Robert Klaassen, Wim JE Tissing, Thomas Lehrnbecher, Julia Chisholm, Hana Hakim, Neil Ranasinghe, Marianne Paesmans, Ian M Hann, Lesley A Stewart

**Correction to:** *British Journal of Cancer* (2016) **114**, 623–630; doi:10.1038/bjc.2016.28; published online 8 March 2016

Upon publication of the above paper in the *British Journal of Cancer*, the authors identified an error in the spelling of one of their names. The publishers would like to apologise for this mistake. Roland A Ammann appears correctly in the author list above.

In addition, the corresponding author was incorrectly designated Professor Robert S Phillips. The correct designation should have been Dr Robert S Phillips.

